# Conditioning regimens in pediatric myeloid malignancies undergoing allogeneic HSCT: a comparative single-center study

**DOI:** 10.3389/fonc.2025.1623636

**Published:** 2025-09-03

**Authors:** Andra D. Marcu, Cristina G. Jercan, Ana M. Bica, Andreea N. Serbanica, Letitia E. Radu, Irina Avramescu, Anda Mocanu, Oana O. Niculita, Delia C. Popa, Cerasela Jardan, Mihaela Dragomir, Andrei Colita, Alina D. Tanase, Anca Colita

**Affiliations:** ^1^ Faculty of Medicine, University of Medicine and Pharmacy Carol Davila, Bucharest, Romania; ^2^ Department of Pediatrics and Bone Marrow Transplantation Unit, Fundeni Clinical Institute, Bucharest, Romania; ^3^ Department of Hematology Laboratory, Fundeni Clinical Institute, Bucharest, Romania; ^4^ Department of Hematology, Coltea Clinical Hospital, Bucharest, Romania; ^5^ Department of Bone Marrow Transplantation, Fundeni Clinical Institute, Bucharest, Romania

**Keywords:** pediatric, myeloid, conditioning regimen, comparison, hematopoietic stem cell transplantation

## Abstract

**Introduction:**

Optimal conditioning regimen for pediatric myeloid malignancies is still subject for debate. This single-center retrospective study compares the efficacy and toxicity profiles of three conditioning strategies, myeloablative conditioning (MAC), reduced-toxicity conditioning (RTC), and reduced-intensity conditioning (RIC), in 59 pediatric patients with myeloid malignancies undergoing allogeneic hematopoietic stem cell transplantation (HSCT).

**Methods:**

Primary objectives evaluated graft versus host disease (GvHD), relapse, overall survival (OS), disease-free survival (DFS), and mortality causes. Secondary endpoints assessed early complications such as mucositis, engraftment kinetics, viral reactivation, and hospitalization duration. A subgroup analysis compared fludarabine- and clofarabine-based RTC regimens.

**Results:**

RTC was associated with significantly lower transfusion needs, faster platelet engraftment and shorter hospitalization. Viral reactivations were more common in RTC and RIC, yet viral control, particularly CMV clearance, seemed more effective in RTC. While one-year OS and DFS were generally comparable across regimens, RTC showed a numerically higher OS, with a possible negative influence on relapse rate for children under 10 years old. Severe acute GvHD was similar across groups, but chronic GvHD tended to occur more frequently in RIC. CR status appeared to influence relapse and mortality patterns, with AML patients transplanted in CR1 experiencing significantly better OS and DFS. Subgroup analysis within RTC (clofarabine vs. fludarabine) revealed promising trends toward improved OS, lower acute GvHD, and reduced relapse-related mortality when using clofarabine.

**Discussions:**

These findings support the use of individualized conditioning strategies in pediatric myeloid malignancies, with RTC emerging as a potentially balanced approach for selected cases.

## 1 Introduction

Pediatric myeloid malignancies are rare and heterogeneous, arising either *de novo* or as secondary disorders. High-risk or relapsed/refractory acute myeloid leukemia (AML), myelodysplastic syndrome (MDS), and juvenile myelomonocytic leukemia (JMML), now classified under myeloproliferative neoplasms (MPN) in WHO 2022, benefit from allogeneic hematopoietic stem cell transplantation (HSCT) as the only curative approach (1). Despite extensive investigation, the optimal conditioning strategy remains undefined, highlighting the need for standardized evaluations to guide protocol refinement and improve outcomes.

A well-balanced choice of conditioning regimen requires taking into consideration both therapeutic benefits and potential adverse effects of high-dose cytotoxic therapy. Graft-versus-leukemia effect is as important as toxicity, graft versus host disease (GvHD) or transplant related mortality. To mitigate the toxicity associated with myeloablative conditioning (MAC), novel regimens have been progressively implemented. Reduced toxicity conditioning (RTC) and reduced intensity conditioning (RIC) have demonstrated lower transplant mortality without increasing the risk of relapse (2). These approaches use either two alkylating agents with non-overlapping non-hematologic toxicities or incorporate synergistic nucleoside analogs to optimize efficacy and reduce adverse effects.

This retrospective study compares MAC, RTC, and RIC regimens in pediatric myeloid malignancies to evaluate differences in toxicity, transplant-related complications and efficacy. Primary endpoints include one-year post-transplant outcomes: acute/chronic GvHD, relapse rates, disease free survival (DFS), overall survival (OS) and mortality. Secondary endpoints assess early post-HSCT events such as oral mucositis (OM), engraftment kinetics, viral reactivation, and hospitalization duration.

## 2 Materials and methods

We conducted a retrospective analysis of 59 pediatric patients diagnosed with myeloid neoplasms treated in the Pediatric Hematology and Oncology and Bone Marrow Transplantation Unit of Fundeni Clinical Institute, Bucharest, Romania between 2005 and 2023. The cohort included a heterogeneous group of diseases: AML, MDS and JMML patients, with diagnoses established according to the international classification criteria applicable at the time of evaluation (1, 3, 4). HSCT was performed for high-risk AML cases achieving first complete remission (CR1) and patients who attained remission following relapse (≥CR2). Secondary disease represents a biologically and clinically distinct entity, arising from antecedent hematologic disorders, prior cytotoxic therapy, or, as recently acknowledged in the WHO 2022 and ICC 2022 classifications, germline predisposition syndromes (1, 5). Pre-transplant bone marrow assessment was conducted to confirm CR status especially in AML patients, based on the minimal residual disease (MRD) evaluation criteria applicable at the time. Pre-transplant CR was defined either as bone marrow blast count below 5% on morphological assessment or as less than 0.1% by flow cytometry.

The first group (MAC, N=23) included BuCy-based regimens, with melphalan added in 8 patients (34.7%), primarily in case of secondary AML, advanced MDS or MPN. Given the melphalan’s known impact on toxicity, this intensified approach may have influenced regimen-related outcomes. Therapeutic drug monitoring (TDM) for busulfan was not available in our clinic. The second group (RTC, N=30) consists of patients receiving either busulfan-based regimens associated with lower-toxicity agents such as fludarabine (FluBu), thiotepa (FluTTBu) or clofarabine (CloFluBu), as well as other reduced toxicity combinations (TTTreoFlu). The third group (RIC, N=10) included patients who either lacked an HLA-matched donor or required urgent transplantation with a reduced intensity profile following chemotherapy. These patients underwent haploidentical HSCT using a thiotepa-melphalan-fludarabine (TMF) conditioning. Detailed protocols are shown in **Table 1**.

**Table 1:** Conditioning regimens protocols.

Conditioning Regimen		Cumulative Dosage						
		Busulfan	Cyclophosphamide	Melphalan	Fludarabine	Thiotepa	Clofarabine	Treosulfan
MAC	BuCy +/- Mel	12.8-19.2 mg/kg IV/PO, 4 days dose (16 doses)	120 mg/m^2^ IV, 2 days dose	140 mg/m^2^ IV, 1 day dose	–	–	–	–
RTC	FluBu	12.8 mg/kg IV/PO, 4 days dose (16 doses)	–	–	180 mg/m^2^ IV, in 6 days dose	–	–	–
	FluTTBu	12.8 mg/kg IV/PO, 4 days dose (16 doses)	–	–	160 mg/m^2^ IV, 4 days dose	8 mg/kg IV, 1 day dose	–	–
	CloFluBu	12.8 mg/kg IV/PO, 4 days dose (4 doses)	–	–	40 mg/m^2^ IV, 4 days dose	–	120 mg/m^2^ IV, 4 days dose	–
	TTTreoFlu	–	–	–	160 mg/m^2^ IV, 4 days dose	10 mg/kg IV, 1 day dose	–	42 g/m^2^ IV, 3 days dose
RIC	TMF	–	–	140mg/m2 IV, 1 day dose	160 mg/m^2^ IV, 4 days dose	5 mg/kg IV, 1 day dose	–	–

MAC, myeloablative conditioning; RTC, reduced-toxicity conditioning; RIC, reduce-intensity conditioning; Bu, busulfan; Cy, cyclophosphamide; Mel, melphalan; Flu, fludarabine; TT, thiotepa; Clo, clofarabine; Treo, treosulfan; TMF, thiotepa-melphalan-fludarabine; mg, milligrams; kg, kilograms; IV, intravenous; PO, per os.

GvHD prophylaxis was donor-adapted: matched sibling donor (MSD) recipients received a calcineurin inhibitor (CNI) with short-course methotrexate; matched unrelated donor (MUD) recipients had either ATG-based prophylaxis or a regimen consisting of tacrolimus, mycophenolate mofetil, and post-transplant cyclophosphamide (PT/Cy); haploidentical recipients uniformly received PT/Cy prophylaxis.

Engraftment was defined as the first of three consecutive days with an absolute neutrophil count exceeding 0.5 × 10^9^/L and the first of seven consecutive days with platelet (PLT) count above 20 × 10^9^/L without transfusion support. Bone marrow or peripheral blood chimerism was assessed using the short tandem repeat method, with donor cell detection of ≥99% considered indicative of complete chimerism.

Post-HSCT monitoring involved the systematic evaluation of early and late transplant-related complications. Major clinical endpoints—DFS, OS, mortality and mortality causes—were analyzed for the entire cohort, within each study group, to assess therapeutic efficacy and outcomes at one-year after HSCT. Acute and chronic GvHD were evaluated based on established consensus criteria (6–8). Post-HSCT viral monitoring is guided by defined thresholds, with sustained cytomegalovirus (CMV) or Epstein-Bar virus (EBV) reactivations marked by viral loads >200 copies/mL or consistent increase across two consecutive measurements. All BK virus (BKV) positive cases were included, as quantitative PCR detection in plasma or urine reliably indicates active replication in pediatric HSCT recipients rather than incidental colonization, and correlates with clinically relevant complications, such as hemorrhagic cystitis. Relapse was defined as hematologic recurrence or MRD re-emergence, as assessed by standardized morphological or immunophenotypic criteria. DFS is the time from HSCT to either relapse of the underlying disease or death from any cause. OS was defined as the duration from the time of transplantation to death from any cause. These metrics are key indicators of HSCT effectiveness across various conditions and patient populations (9, 10).

This study was approved by the institutional ethics committee on February 6, 2025, and conducted in accordance with the local legislation and institutional requirements. Written informed consent was obtained from legal guardians at the time of diagnosis and during each subsequent hospital admission. Consent covered both diagnostic and therapeutic procedures as well as participation in scientific research. All clinical data were anonymized and extracted from institutional medical records and databases.

### 2.1 Statistical analysis

Data analysis was performed using IBM SPSS Statistics version 25 (IBM Corp., Armonk, NY, USA). Categorical variables were represented as absolute counts and percentages. Associations between categorical variables were assessed using Pearson’s Chi-squared or Fisher’s Exact Test. The Shapiro-Wilk test confirmed that all continuous variables did not follow a normal distribution. Continuous variables were expressed as median, minimum, and maximum. Differences between groups were analyzed using the Kruskal-Wallis or Mann–Whitney U test and visualized with boxplots. Correlations between two continuous variables were assessed using Spearman’s rho and depicted in scatter plot. Survival outcomes, including overall survival (OS) and disease-free survival (DFS), were estimated at one year and compared using cross-tabulations and chi-square tests. A two-sided p-value <0.05 was considered statistically significant. Boxplot and scatterplot visualizations were used for illustration.

## 3 Results

The study cohort included 59 pediatric patients undergoing allogeneic HSCT for myeloid malignancies, four of which received second transplant, yielding a total of 63 datapoints. The general characteristics of the cohort are presented in **Table 2**. Overall sex ratio was balanced (1:1), and the median age at transplant was 10 years (range 1–17 years). Age distribution varied across conditioning regimens with 69.6% of MAC patients <10 years compared to only 20.0% in RIC (*p* = 0.009, Pearson Chi-Square; *p* = 0.020, Fisher’s Exact Test).

**Table 2:** General characteristics of the cohort.

Variable		Overall (N=63)	MAC (N=23)	RTC (N=30)	RIC (N=10)	*p*-value	MAC *vs* RTC	RTC *vs* RIC	RIC *vs* MAC
Gender	Male Female	33 (53.4%) 30 (47.6%)	10 (43.5%) 13 (56.5%)	17 (56.7%) 13 (43.3%)	6 (60.0%) 4 (40.0%)	0.553	0.341	0.853	0.383
Age at HSCT	Median	10 (1,17)	7 (1,17)	10 (1, 17)	11.5 (1, 16)	0.156	0.314	0.363	0.034
	≤ 10 yo ≥ 10 yo	31 (49.2%) 32 (50.8%)	16 (69.6%) 7 (30.4%)	13 (43.3%) 17 (56.7%)	2 (20.0%) 8 (80.0%)	**0.022**	0.057	0.187	**0.020**
Diagnostic	AML MDS JMML	45 (71.4%) 10 (15.9%) 8 (12.7%)	15 (65.2%) 3 (13.0%) 5 (21.7%)	22 (73.3%) 6 (20.0%) 2 (6.7%)	8 (80.0%) 1 (10.0%) 1 (10.0%)	0.510	0.255	0.747	0.670
Disease origin	*De novo* Secondary	47 (74.6%) 16 (25.4%)	16 (69.6%) 7 (30.4%)	24 (80.0%) 6 (20.0%)	7 (70.0%) 3 (30.0%)	0.644	0.382	0.512	0.980
Pre-HSCT AML status	CR1 ≥ CR2	34 (75.6%) 11 (24.4%)	10 (66.7%) 5 (33.3%)	20 (90.9%) 2 (9.1%)	4 (50.0%) 4 (50.0%)	0.306	0.064	**0.013**	0.435
Type of HSCT	MSD MUD Haplo	18 (28.6%) 34 (54.0%) 11 (17.5%)	11 (47.8%) 11 (47.8%) 1 (4.3%)	7 (23.3%) 23 (76.7%) 0 (0.0%)	0 (0.0%) 0 (0.0%) 10 (100.0%)	**<0.001**	0.071	**<0.001**	**<0.001**
Stem Cell Source	BM PBSC	9 (14.3%) 54 (85.7%)	6 (26.1%) 17 (73.9%)	3 (10.0%) 27 (90.0%)	0 (0%) 10 (100.0%)	0.094	0.122	0.298	0.074
GvHD prohylaxis	CSA/Tacro, short MTX ATG, CSA, short MTX PT/Cy, Tacro, MMF Other	18 (28.6%) 16 (25.4%) 28 (44.4%) 1 (1.6%)	11 (47.8%) 10 (43.5%) 1 (4.3%) 1 (4.3%)	7 (23.3%) 6 (20.0%) 17 (56.7%) 0 (0.0%)	0 (0.0%) 0 (0.0%) 10 (100.0%) 0 (0.0%)	**<0.001**	**0.001**	**0.040**	**<0.001**

vs, versus; HSCT, hematopoietic stem cell transplantation; yo, years old; AML, acute myeloid leukemia; MDS, myelodysplastic syndrome; JMML, juvenile myelomonocytic leukemia; CR, complete remission; MSD, matched sibling donor; MUD, matched unrelated donor; Haplo, haploidentical donor; BM, bone marrow; PBSC, peripheral blood stem cells; GvHD, graft versus host disease; CSA, cyclosporine; Tacro, tacrolimus; MTX, methotrexate; ATG, anti-thymocyte globulin. Note: Bold values indicate statistically significant results (p < 0.05).

Diagnoses distribution (AML, MDS, JMML) and disease origin (*de novo* vs secondary) was comparable across groups. The study cohort consisted of 45 HSCT procedures for AML with 24.4% achieving ≥ CR2 before transplant, 10 cases of MDS and 8 cases of JMML. Secondary AML was identified in 25.4% of patients. A significant difference was observed in the distribution of pre-transplant disease status between RTC and RIC groups (*p* = 0.013), with a higher proportion of RTC patients undergoing HSCT in CR1 (90.9%) compared to RIC (50.0%), where more patients in ≥CR2 were included (50.0% vs 9.1%). This discrepancy likely reflects both the clinical urgency to proceed to transplant in patients with relapsed disease and the constraints of donor availability, as RIC was exclusively used in haploidentical settings.

Donor distribution differed significantly across regimens (*p* < 0.001), with all RIC recipients undergoing haploidentical grafts, whereas MAC and RTC cohorts included a mixture of MSD, MUD and a smaller proportion of haploidentical donors. Peripheral blood stem cells (PBSC) were the predominant stem cell source across all groups (85.7% overall). GvHD prophylaxis varied significantly across conditioning groups (*p* < 0.001), reflecting underlying donor patterns. Notably, all RIC recipients received PT/Cy-based prophylaxis, aligning with exclusive haploidentical transplantation in this group. Most MAC recipients (91.3%) were treated with CNI-based regimens, while the RTC group exhibited a hybrid approach, with 56.7% receiving PT/Cy. These patterns highlight the interplay between conditioning intensity, donor selection, and immunosuppressive strategies.

**Figure 1:**
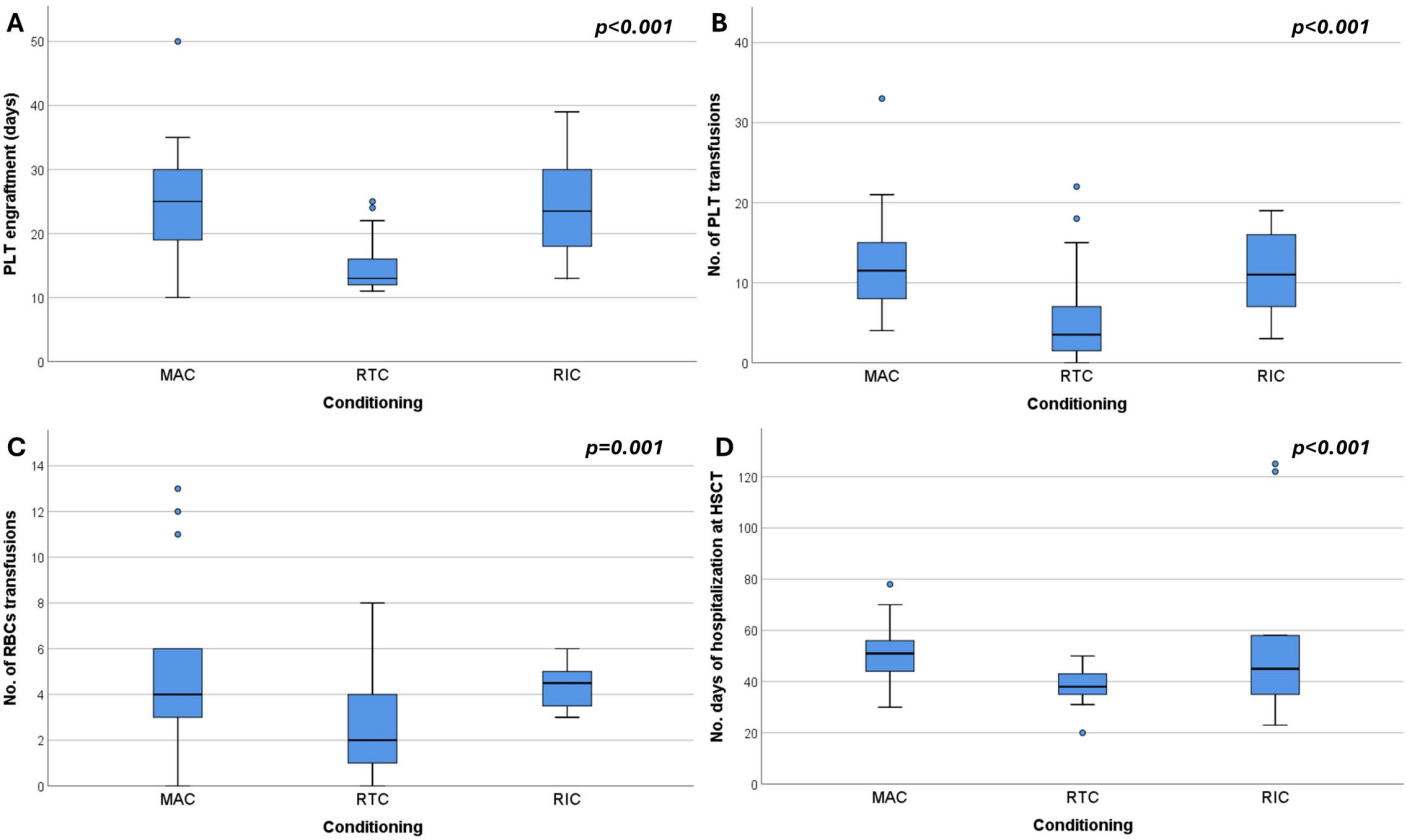
Box plots showing **(A)** platelet (PLT) engraftment time, **(B)** number of PLT transfusions, **(C)** number of red blood cell (RBC) transfusions, and **(D)** number of hospitalization days at HSCT, stratified by conditioning regimen: MAC (myeloablative conditioning), RTC (reduced-toxicity conditioning), and RIC (reduced-intensity conditioning).

**Figure 2:**
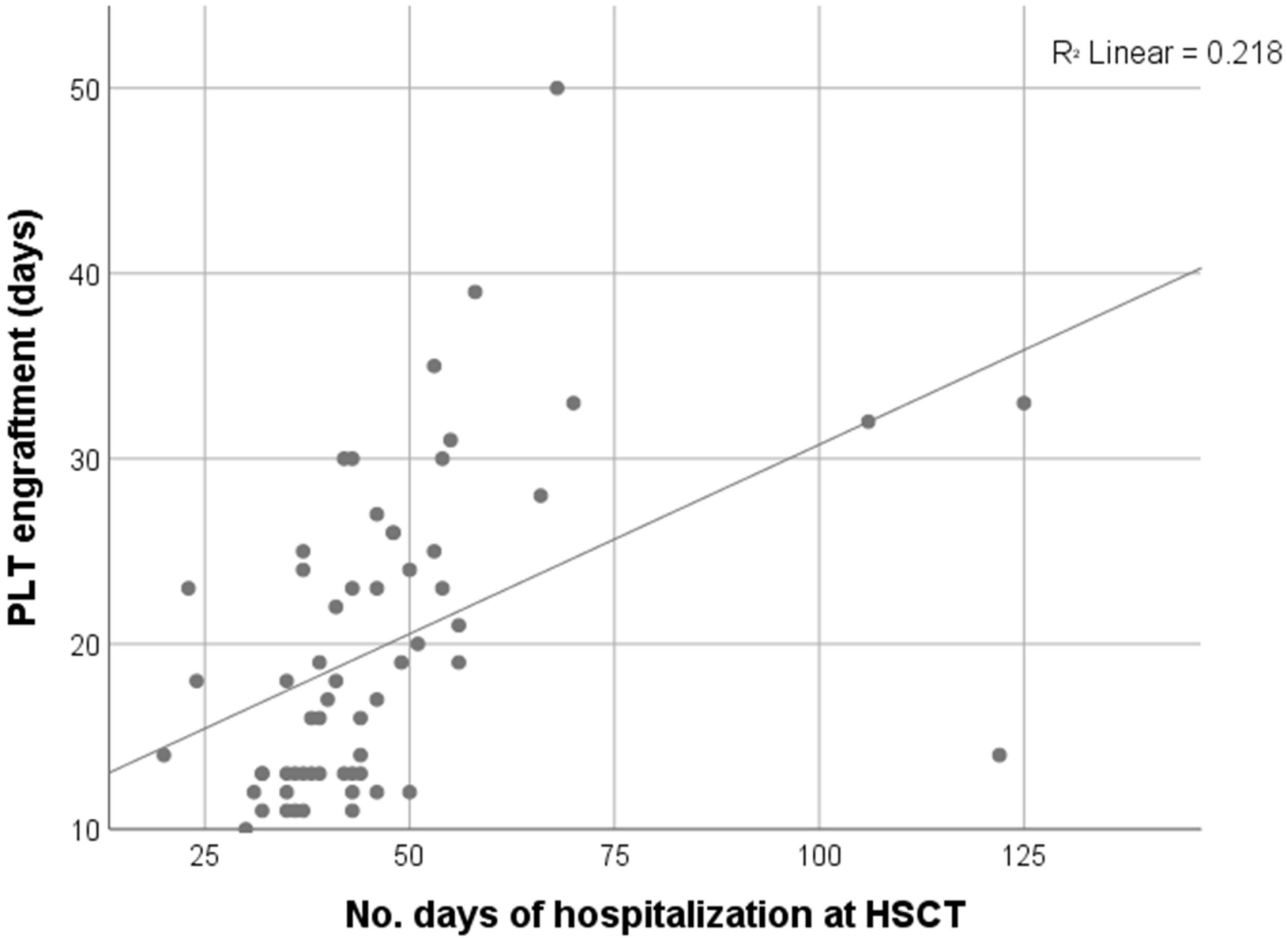
Moderate to strong positive correlation between PLT engraftment and length of hospitalization (Spearman’s rho = 0.616, *p* < 0.001, N = 60).

Comparative analysis further showed that patients receiving RTC required significantly less transfusion support. The median number of erythrocyte transfusions was 2 units (0–8) in RTC compared to 4 units (range 0–31) in MAC and 5 units (3–50) in RIC, with a significant difference between RTC and the other two regimen strategies (*p* = 0.010, *p* = 0.005 respectively). Similarly, platelet transfusion requirements were lowest in RTC (4 units) compared to MAC (12 units) and RIC (15 units), with RTC vs MAC or RIC reaching statistical significance (*p* < 0.001, *p* = 0.009 respectively). Platelet engraftment occurred significantly earlier in the RTC (median 13 days, range 11–25) compared to MAC (25 days, range 10–50) or RIC (MAC vs RTC, *p* < 0.001; RTC vs RIC, *p* < 0.002). Neutrophil engraftment, however, did not differ significantly among the groups (median ~20 days, *p* = 0.309). Boxplot analyses support these findings, indicating trends toward reduced transfusion demand and earlier platelet recovery in RTC (**Figure 1**). Moreover, MAC patients had prolonged hospital stays (median 53 days) compared to RTC (38 days, *p* < 0.001), with RIC showing intermediate durations (45 days). A significant positive correlation was observed between time to platelet engraftment and hospitalization length (Spearman’s ρ = 0.616, *p* < 0.001; **Figure 2**), suggesting that delayed hematologic recovery contributes to the extended inpatient care (**Figure 2**).

Oral mucositis (OM) was documented in all 63 evaluable cases, with significantly greater severity in the MAC group, where grade III–IV OM occurred in 73.9% of patients, compared to 23.3% in RTC (*p* = 0.001) and 40.0% in RIC (*p* = 0.063). *Clostridioides difficile* infection (CDI) correlated with higher-grade OM was observed in 83.3% of CDI-positive patients versus 35.3% of CDI-negative patients (*p* = 0.003). General digestive colonization, however, showed no significant association with OM severity (*p* = 0.212). A modest linear association was noted between the number of gut pathogens and mucositis grade (*p* = 0.035).

Viral reactivation occurred in 62.9% of patients, with multiple reactivations observed in 19%, primarily in the RIC group. While overall reactivation rates did not significantly differ by regimen (*p* = 0.072), RTC was associated with a higher rate compared to MAC (73.3% vs 43.5%; *p* = 0.028; confirmed by Fisher’s exact test, *p* = 0.047). BKV reactivation occurred in 47.6% of all patients, notably more frequent in RTC and RIC compared to MAC (*p* = 0.008 and *p* = 0.032, respectively). Although more common with PT/Cy prophylaxis (57.1% vs. 38.2%), the association was not statistically significant (*p* = 0.138). BKV resolution occurred in 86.7% of the overall affected patients. CMV reactivation occurred in 33.3% of patients, with no significant difference across regimens (*p* = 0.229). Multiple reactivations were most frequent in RIC (50%) and least in RTC (16.7%, *p* = 0.067). Despite this, MAC patients had the highest viral loads (median 15,000 vs 2,870 in RTC and 2,509 in RIC) and lowest resolution rates (44.4%), significantly worse than RTC (91.7%, *p* = 0.018). Further compared to combined RTC/RIC, MAC group confirmed lower clearance (*p* = 0.028) and higher viral burden, validated by Kruskal–Wallis (*p* = 0.043) and *post-hoc* RTC vs MAC (*p* = 0.041).

Post-HSCT CR was achieved in 90.0% of patients (*p* = 0.982), with uniformly high donor chimerism across groups (*p* = 0.068), indicating robust engraftment and reliability of sustained donor hematopoiesis across diverse conditioning protocols.

One-year outcome analysis (**Table 3**) excluded two patients who experienced graft failure and required second transplant. Grade III-IV acute GvHD (aGvHD) occurred in 20.3% of overall cohort and the lack of statistical differences between groups (*p*=0.312) suggests comparable risk for advanced aGvHD. When analyzing all cases of aGvHD, including mild symptoms, skin involvement was more frequent in RIC (8/10) compared to MAC (9/21, *p* = 0.05) or RTC (16/28, *p* = 0.197). Gastrointestinal aGvHD affected 22.1% of cases (13/59), with the highest incidence in RIC (5/10, 50.0%), followed by MAC (5/21, 23.8%, RIC vs MAC *p*= 0.144) and lowest in RTC (3/28, 10.8%, RIC vs RTC *p* = 0.008). Resolution rates for skin (80–100%, *p* = 0.30) and gut (33.3–50%, *p* = 0.850) aGvHD were comparable across groups. CDI was strongly associated with persistent gut aGvHD (0% vs. 66.7% resolution, *p* = 0.022), indicating a potential interaction between microbial disruption and GvHD outcomes. Severe cGvHD was highest in the RIC group (20.0%) and significantly more frequent than in MAC (*p* = 0.034), indicating increased first-year risk with RIC.

**Table 3:** Comparative analysis on strategies of conditioning regimens.

Variable	Overall (N=63)	MAC (N=23)	RTC (N=30)	RIC (N=10)	P-value	MAC vs RTC	RTC vs RIC	RIC vs MAC
OM Grade I-II Grade III-IV	35 (55.6%) 28 (44.4%)	6 (26.1%) 17 (73.9%)	23 (76.7%) 7 (23,3%)	6 (60.0%) 4 (40.0%)	**0.001**	**<0.001**	0.307	0.063
Erythrocyte transfusion	4 (0, 50)	4 (0, 31)	2 (0, 8)	5 (3, 50)	**0.001**	**0.010**	**0.005**	1.000
PLT transfusion	8 (0, 71)	12 (4, 57)	4 (0, 25)	15 (7.25, 22)	**<0.001**	**<0.001**	**0.009**	1.000
Neutrophil engraftment	20 (12, 34)	21 (12, 34)	20 (13, 30)	13 (3, 71)	0.309	0.340	0.155	0.509
PLT engraftment	18 (10, 50)	25 (10, 50)	13 (11, 25)	23.5 (13, 39)	**<0.001**	**<0.001**	**<0.002**	1.000
*Clostridioides Difficile* infection	7 (11.1%)	3 (13%)	2 (6.7%)	2 (20.0%)	0.879	0.429	0.222	0.610
Viral reactivation Yes Multiple CMV BKV	39 (62.9%) 12 (19.0%) 21 (33.3%) 30 (47.6%)	10 (43.5%) 4 (17.4%) 9 (39.1%) 5 (21.7%)	22 (73.3%) 4 (13.3%) 7 (23.3%)19 (63.3%)	7 (70.0%) 4 (40.0%) 5 (50.0%) 6 (60.0%)	0.072 0.172 0.229 **0.008**	**0.028** 0.683 0.214 **0.003**	0.838 0.068 0.111 0.850	0.161 0.164 0.198 **0.032**
Post-HSCT CR	54 (90.0%)	20 (90.9%)	25 (89.3%)	9 (90.0%)	0.982	0.849	0.950	0.935
No. days of hospitalization	43 (20, 125)	53 (30, 106)	38 (20, 50)	45 (23, 125)	**<0.001**	**<0.001**	0.279	0.418
1-year outcomes	Overall (N=61)	MAC (N=22)	RTC (N=29)	RIC (N=10)	*P-*value	MAC *vs* RTC	RTC *vs* RIC	RIC *vs* MAC
Grade III-IV aGvHD	12 (20.3%)	5 (23.8%)	5 (17.8%)	2 (20.0%)	0.312	0.610	0.880	0.810
Severe cGvHD	4 (6.7%)	0 (0.0%)	2 (7.1%)	2 (20.0%)	0.322	0.211	0.254	**0.034**
Overall survival	46 (75.4%)	15 (68.2%)	23 (79.3%)	8 (80.0%)	0.796	0.368	0.960	0.490
Disease free survival	37 (60.7%)	14 (63.3%)	18 (62.1%)	5 (50.0%)	0.748	0.912	0.502	0.728
Mortality Relapse GvHD Infection Others	15 (24.6%) 5 (8.2%) 4 (6.6%) 3 (4.9%) 3 (4.9%)	7 (31.8%) 2 (9.1%) 3 (13.6%) 1 (4.5%) 1 (4.5%)	6 (20.7%) 3 (10.3%) 0 (0.0%) 1 (3.4%) 2 (6.9%)	2 (20.0%) 0 (0.0%) 1 (10.0%) 1 (10.0%) 0 (0.0%)	0.603 0.579 0.133 0.707 0.682	0.368 0.880 **0.040** 0.841 0.726	0.960 0.289 0.083 0.417 0.395	0.490 0.327 0.771 0.555 0.496

OM, oral mucositis; PLT, platelets; GvHD, graft versus host disease; CMV, cytomegalovirus; BKV, BK virus; HSCT, hematopoietic stem cell transplantation; CR, complete remission; No, number; aGvHD, acute graft versus host disease; cGvHD, chronic graft versus host disease. Note: Bold values indicate statistically significant results (p < 0.05).

Mean survival at one year was 9.4 months (95% CI: 8.4–10.5). OS was numerically higher in RIC (80%) and RTC (79.3%) compared to MAC (68.2%), with no significant differences (global *p*=0.796; all pairwise comparisons >0.3). DFS showed no clear advantage among regimens (*p* = 0.748). Overall mortality was 24.6%, highest in the MAC group (31.8%, *p* = 0.603). GvHD-related mortality occurred exclusively in MAC (13.6%), while relapse- and infection-related deaths were comparable across groups. The small RIC subgroup (N = 10) may limit result reliability.

Further one-year relapse rates varied by regimen (MAC: 14.3%, RTC: 28.6%, RIC: 30.0%) without reaching significance (*p* = 0.446). In children under 10 years, relapse was more frequent after RTC (6/8) than MAC (2/3) or RIC (1/3), with a borderline trend (*p* = 0.070; linear trend *p* = 0.026) and significant correlation (Spearman’s ρ = 0.414, *p* = 0.029). No differences were seen in patients ≥10 years (*p* = 0.839). When extended to the full follow-up period, this pattern persisted, with a near-significant overall association (*p* = 0.079).

Accounting for disease origin, this study included 26.2% patients with secondary and 73.7% with *de novo* malignancies. One-year OS, DFS, and GvHD rates were similar between groups. Relapse-related deaths occurred exclusively in *de novo* cases (11.4% vs 0%; *p* = 0.147), as well as severe cGvHD (9.1% vs 0%; *p* = 0.423). Infection- and GvHD-related mortality remained low and comparable.

Analysis of the AML procedures (N= 43) shows that achieving CR1 prior to HSCT promotes superior outcomes compared to ≥CR2. One-year OS differed significantly across all groups (CR1: 93.5%, ≥CR2: 63.6%; χ² = 6.216, *p* = 0.045), as did DFS (CR1: 80.6%, ≥CR2: 36.4%; χ² = 7.991, *p* = 0.018). Survival was highest in CR1 patients receiving RTC (94.7%) and RIC (100%), though differences by regimen were not significant (*p*= 0.575 and *p*=0.548 respectively). Relapse and infection-related mortality were higher in ≥CR2, with infection-related deaths reaching significance (*p* = 0.047). GvHD-related mortality was minimal and confined to ≥CR2, with no differences in severe GvHD incidence.

Among 29 evaluable patients receiving RTC, administration of clofarabine-based regimens (N=17, cumulative Clo dose 120mg/m^2^) demonstrated higher one-year OS (88.2% vs 66.7%) and DFS (64.7% vs 58.3%) compared to fludarabine-based regimens (N=12, cumulative Flu dose ≥160 mg/m^2^), though differences were not statistically significant (*p* = 0.198 and *p* = 1.000 respectively). Grade III–IV aGvHD occurred more frequently in the Flu-based group (25.0% vs 11.8%), with a borderline association (χ² = 5.158, *p* = 0.076; linear trend *p* = 0.042). Severe cGvHD was observed only in the clofarabine group (11.8%, *p* = 0.325). Relapse-related mortality was higher in the Flu-based group (16.7%) compared to Clo-based (5.9%), with one additional infection-related death (8.3%) in the Flu arm. However, these differences were not statistically significant and may reflect sample-related variability rather than a true regimen-related effect.

## 4 Discussions

The optimal conditioning regimen for myeloid neoplasms is still open for debate and investigation, especially because studies in pediatric population lack robustness due to less standardized protocols.

MAC regimens—typically comprising busulfan and high-dose cyclophosphamide (BuCy), with melphalan added in select high-risk cases—remain a cornerstone to pediatric myeloid transplant protocols due to their capacity for reliable engraftment and sustained leukemia control. However, their use is limited by substantial toxicity, particularly in older children, despite advances in therapeutic drug monitoring (TDM) and supportive care (11–17). Prior studies have identified younger age as an independent predictor of relapse and reported elevated HSCT-related mortality in children over 10 years receiving MAC (12, 18). In our cohort, conditioning selection reflected clinical judgment: MAC was primarily used in younger patients, while RIC was preferred for older children with cumulative toxicity and urgent transplant indication.

RTC regimens use tailored dosing and agents like fludarabine, clofarabine, and/or treosulfan to minimize regimen-related toxicity while preserving antileukemic efficacy. The substitution of cyclophosphamide with fludarabine has shown comparable outcomes (19). However, there are reports of shorter 2-year OS with BuFlu compared to BuCy (61% vs 71%, *p* = 0.01), mainly due to poorer post-relapse survival, independent of pre-HSCT disease status (20). Clofarabine, with its enhanced synergy and tolerability, has been increasingly adopted in CloFluBu regimens, associated with lower rates of aGvHD, especially cutaneous and hepatic toxicity (12). Treosulfan offers a more favorable safety profile than busulfan, with improved early gastrointestinal tolerability, reduced relapse and transplant-related mortality (21–24). RTC aims to preserve efficacy while minimizing MAC-related toxicity, with the goal of improving survival outcomes and quality of life.

In this study, MAC was linked to a higher toxicity burden compared to RTC, reflected in higher transfusion needs, delayed platelet engraftment, longer hospitalization, and more frequent grade III–IV OM. RIC showed toxicity levels comparable to MAC, suggesting reduced intensity does not always lessen supportive care demands. In contrast, RTC demonstrated a more favorable toxicity profile without compromising early outcomes. The strong correlation between delayed engraftment and hospitalization (Spearman’s ρ = 0.616, *p* < 0.001) highlights the clinical impact of hematologic recovery.

Consistently, our findings reveal a strong link between conditioning intensity and mucosal toxicity, with MAC significantly associated with severe and prolonged OM. This barrier injury facilitates microbial translocation, increasing the risk of systemic infections and inflammatory complications after HSCT (25–27). While not statistically conclusive, the observed trend between multiple GI pathogens and severe mucositis may reflect cumulative microbial burden, reinforcing the importance of microbial surveillance and early intervention. Furthermore, *Clostridioides difficile* infection (CDI) was specifically associated with grade III–IV OM (*p* = 0.003) and non-resolution of gastrointestinal aGvHD (*p* = 0.022), suggesting a pathogenic role beyond colonization and a bidirectional interaction between microbial dysbiosis and immune-mediated gut damage (28). This interaction has been associated with reduces OS, increased transplant- and infection-related mortality, with aGvHD itself identified as an independent risk factor for post-engraftment CDI (29). Gastrointestinal aGvHD was most frequent in RIC and least common in RTC (*p* = 0.008), indicating a potential gut-protective effect of RTC. These findings collectively show the interplay between conditioning-related toxicity, microbial imbalance, and GvHD expression, highlighting the critical role of barrier protection and infection control in optimizing transplant outcomes. Regarding cGvHD, RIC group showed higher incidence of severe disease compared to MAC (*p* = 0.034), along with increased rates of skin involvement. However, these findings should be interpreted cautiously given the limited sample size. Several studies show that in haploidentical HSCT, RIC with PT/Cy enables timely transplantation for high-risk pediatric patients without matched donors, offering survival outcomes comparable to MAC, with reduced toxicity and reliable engraftment (30, 31). While PT/Cy reduces GvHD by modulating T-cell alloreactivity, it may delay immune reconstitution and increase viral susceptibility (32, 33). In our study, any detectable BKV was considered clinically relevant. Although often self-limiting, BKV is known for its hemorrhagic cystitis risk and may reflect subclinical immune dysfunction, supporting a low threshold for monitoring, particularly in patients receiving PT/Cy. BKV reactivation was significantly more common in RTC versus MAC (63.3% vs. 21.7%, *p* = 0.008), potentially reflecting the use of PT/Cy in some RTC recipients (17/30). However, resolution rates remained high across all regimens, suggesting that despite higher initial susceptibility, RTC and RIC do not impair BKV clearance when appropriately managed. Furthermore, RTC showed superior CMV control, with higher resolution rates (91.7% vs. 44.4%, *p* = 0.018) and lower viral loads, indicating good preservation of immune function. In contrast, MAC was associated with higher CMV burden and impaired clearance, possibly reflecting greater immune suppression and mucosal injury, facilitating viral reactivation and systemic spread.

Our survival findings suggest that lower-intensity regimens may offer comparable early survival outcomes to MAC regimens. RTC preserved comparable one-year OS (79.3%) and DFS (62.1%) relative to MAC and RIC, likely due to reduced toxicity and better early transplant tolerance. Although individual mortality causes did not reach statistical significance, a trend toward increased GvHD-related mortality for MAC patients was observed, with 2/3 patients having received melphalan as part of conditioning. Among the eight patients diagnosed with JMML, only one achieved long-term survival following a second allogeneic transplant, ultimately attaining complete remission with documented molecular clearance. Four patients died due to post-transplant relapse. These outcomes highlight the particularly poor prognosis associated with JMML in our cohort, consistent with existing literature. Furthermore, age-stratified analysis revealed a notable connection between age and relapse risk: RTC was associated with higher relapse rates in children under 10 years, with a significant linear association, while relapse in older patients (≥10 years) appeared unaffected by conditioning intensity. This age-dependent pattern, which persisted over time, underscores the need for age-adapted conditioning strategies and supports prospective validation in larger cohorts.

Despite the traditionally poorer prognosis associated with secondary myeloid malignancies (34), when stratified by disease origin, our study analysis did not reveal significant differences in key post-transplant outcomes when compared with *de novo* cases. This suggests that HSCT may mitigate some of the adverse biology typically linked to secondary disease, while absence of relapse-related mortality in this group may reflect differences in disease kinetics or more intensive pre-transplant selection. Therefore, pre-transplant disease status is a key prognostic factor in pediatric AML. While morphological CR encompasses a wide spectrum of residual leukemic burden, MRD is a well-established predictor of post-HSCT outcomes (35). Studies suggest that MAC offers superior relapse control and survival in patients with evidence of residual disease (36), whereas OS appears comparable between MAC and RIC in patients achieving CR at transplant (37, 38). Our findings reinforce the prognostic value of early disease control prior to HSCT in pediatric AML, with significantly superior one-year OS (93.5%) and DFS (80.6%) for patients in CR1 compared to those in ≥CR2 (OS: 63.6%, DFS: 36.4%; *p* < 0.05). Despite similar GvHD rates, infection-related mortality was higher in ≥CR2 patients (18.2%), reflecting their increased vulnerability and the need for enhanced supportive care beyond first remission. Busulfan exposure may contribute to outcome disparities, as both under- and overexposure adversely affect transplant success (13). Optimized busulfan-fludarabine regimens have shown benefit in CR2 AML, supporting individualized dosing and pharmacokinetic-guided conditioning (39).

Further subgroup analysis within RTC group suggests a potential clinical advantage for clofarabine when compared with fludarabine-based conditioning, reflected in higher one-year OS (88.2% vs 66.7%) and lower incidence of severe aGvHD (11.8% vs 25.0%), although not statistically significant. The trend toward increased relapse- and infection-related mortality in the fludarabine group may reflect underlying differences in immunosuppressive toxicity or disease risk. Notably, clofarabine appears to be a promising RTC backbone in pediatric HSCT, warranting further prospective investigation.

This study is limited by its retrospective, single-center design, variability in baseline disease characteristics, donor type and GvHD prophylaxis across conditioning groups. Heterogeneity within the RTC group has been partially addressed through subgroup analysis. Differences in patient distribution, particularly within the RIC/haplo cohort, may reflect both small sample size and center-specific practice. The relatively small RIC cohort limits the statistical power for subgroup analyses, constraining the ability to draw definitive conclusions regarding outcomes in this group. Moreover, multivariate analysis was not feasible due to the small sample size (especially in the RIC cohort), unequal group distribution, few outcome events, and skewed distributions of continuous variables. Another key limitation is the lack of busulfan TDM, which may have introduced variability in toxicity and efficacy. This could have influenced outcomes, particularly in MAC and RTC groups where busulfan plays a central role. Nonetheless, this structured comparison provides valuable insight into conditioning-related outcomes in pediatric HSCT, particularly given the challenges of studying rare diseases with limited patient populations.

## 5 Conclusions

The optimal conditioning regimen for pediatric myeloid malignancies remains a topic of ongoing investigation. These findings highlight the importance of a risk- and age-adapted approach to conditioning in pediatric setting. This study suggests that reduced-toxicity regimens, particularly those incorporating clofarabine, may offer a favorable balance between efficacy and tolerability in selected patients, most notably in children over 10 years transplanted in first complete remission. While early outcomes are encouraging, long-term efficacy and relapse risk must be further evaluated through prospective, multicenter studies.

## Data Availability

The raw data supporting the conclusions of this article will be made available by the authors, without undue reservation.
